# Evaluation of the metagenomic next-generation sequencing performance in pathogenic detection in patients with spinal infection

**DOI:** 10.3389/fcimb.2022.967584

**Published:** 2022-10-27

**Authors:** Yi Zhang, Jinmei Chen, Xiaoli Yi, Zhiheng Chen, Ting Yao, Zhenghao Tang, Guoqing Zang, Xuejie Cao, Xiaofeng Lian, Xiaohua Chen

**Affiliations:** ^1^ Department of Infectious Diseases, Shanghai Jiao Tong University Affiliated Sixth People’s Hospital, Shanghai, China; ^2^ Genoxor Medical Science and Technology Inc., Shanghai, China; ^3^ Department of Orthopedics, Shanghai Jiao Tong University Affiliated Sixth People’s Hospital, Shanghai, China

**Keywords:** spinal infection, microbial culture, pathogenic detection, diagnosis, metagenomic next-generation sequencing (mNGS)

## Abstract

Spinal infection is a rarely occurred pathology, whose diagnosis remains a major challenge due to the low sensitivity of culturing techniques. Metagenomic next-generation sequencing (mNGS) is a novel approach to identify the pathogenic organisms in infectious diseases. In this study, mNGS technology was adopted for pathogenic detection in spinal infection from the tissue and pus samples. Additionally, the diagnostic performance of mNGS for spinal infection was evaluated, by comparing it with that of the conventional microbial culture, with the histopathological results as the gold standard. Overall, 56 samples from 38 patients were enrolled for mNGS testing, and 69 samples were included for microbial culture. 30 patients (78.95%) were identified to be positive by the mNGS method, which was higher than that of microbial culture (17, 44.74%). The sensitivity and specificity of mNGS with pus samples were 84.2% and 100.0%, respectively, which outperformed those of microbial culture (42.1% and 100.0%). The pathogen identification results were applied to medication guidance, and all 38 patients experienced favorable outcomes at three months, followed-up post-treatment, without any adverse effects. These findings proved that mNGS was superior to microbial culture in pathogenic identification of the spinal infection, thereby showing great promise in guiding drug administration and improving clinical outcomes.

## Introduction

Infections occurring in the vertebral bodies, intervertebral discs, and surrounding soft tissues are all classified as spinal infections (Lener et al., 2018). Pathogens can be directly inoculated during spinal surgery or contiguously spread by adjacent site infection (Tsantes et al., 2020). Spinal infection symptoms are generally nonspecific and range from back pain to fever, spinal deformation, instability, and even death (Cornett et al., 2016; Nagashima et al., 2018), which hampers the diagnosis of spinal infection. In the early stage of diagnosis, magnetic resonance imaging (MRI) is usually applied to exclude other non-infectious spinal diseases (Fucs et al., 2012). Generally, microbial culture is the primary method to distinguish the microorganisms in bone and joint infections; however, the positive rate is only around 40% to 70% due to antibiotic treatment, fastidious microorganisms, and biofilm formation (Huang et al., 2020).

Molecular-level analysis technologies, such as polymerase chain reaction (PCR), have also been employed in the etiological diagnosis of spinal infection (Merino et al., 2012). However, it requires empirical speculation of potential pathogens by clinical symptoms, which limits the detection scope, especially for rare pathogens and complex infections. Metagenomic next-generation sequencing (mNGS), as a culture-independent and unbiased analysis method, has attracted wide attention. Most studies have demonstrated that mNGS has a similar sensitivity to specific PCR assays (Miller and Chiu, 2021). Moreover, mNGS has been proven capable of identifying more potential pathogens than conventional methods, especially for rare, indistinguishable, and complex pathogens (Han et al., 2019).

The usability of mNGS for diagnosing osteoarticular infection has been evaluated by comparing it with other standard methods. As reported, mNGS improved the diagnosis of osteoarticular infection from abscess specimens, with a greater pathogen detection rate than conventional culture-based methods (Zhao et al., 2020). In the prosthetic joint infection, a higher positive rate has been observed by mNGS than microbial culture, with even higher sensitivity and specificity when the culture results were negative (Thoendel et al., 2018). In addition, mNGS has been proven to be more sensitive than broad-range PCR for detecting prosthetic joint infection in joint fluid and be capable of identifying more pathogens in polymicrobial and fungal infections (Wang et al., 2020). In spinal-related infection, disseminated tuberculosis has been successfully diagnosed using mNGS from the resected sample from spinal surgery (Ye et al., 2021).

Moreover, the potential of mNGS in the etiological diagnosis of spinal infection has been revealed, achieving a sensitivity and specificity of 70.3% and 75.0%, respectively (Ma et al., 2022). Therefore, this study was conducted to evaluate the diagnostic performance of mNGS in pathogenic detection involved in spinal infection, by comparing it with that of the traditional microbial culture. Furthermore, the potential clinical benefits of mNGS in medication guidance and improving clinical outcomes were also analyzed, as well as the choice of optimal sample types for mNGS testing.

## Materials and methods

### Patient enrollment, clinical assessment, and sample collection

From January 1st to December 31st, 2021, a cohort of patients with spinal infection was admitted to the Department of Infectious Diseases in Shanghai Jiao Tong University Affiliated Sixth People’s Hospital. Spinal infection is an infectious disease affecting the vertebral body, the intervertebral disc, and the adjacent paraspinal tissue. All subjects with a suspected spinal infection underwent surgical treatment. The collected tissue and pus samples were histopathologically examined and sent for the mNGS and microbial culture for etiological examination. Only those finally diagnosed with spinal infection were enrolled and analyzed in this manuscript. Suspicion of spinal infection was based on the patients’ complaints, clinical symptoms, laboratory examinations, and imaging findings, including MRI and computed tomography (CT). Diagnosis of spinal infection was based on the above results, combined with histopathological analysis of infected tissues. The most common complaints of patients with spinal infection are back or neck pain, depending on the location of the infection (Tsantes et al., 2020). Laboratory examinations include white blood cell (WBC) count and concentrations of inflammatory factors such as erythrocyte sedimentation rate (ESR) and C-reactive protein (CRP). MRI remains the most reliable method to diagnose spinal infection, and CT is devoted to precisely detecting bony changes and bone necrosis.

In case of doubt, surgical treatment should be considered. All patients in the present study underwent surgical intervention, percutaneous transforaminal endoscopic debridement and drainage (Chen et al., 2022). The generated tissue and pus samples were collected during surgery, and then pathological examinations were conducted with the infected tissues. At the same time, their samples were sent for pathogenic identification within two hours to identify the causative infectious organisms. Finally, 38 patients were diagnosed with spinal infection and had not received any antimicrobial therapy before sample collection. Clinical and demographic data of these patients were collected from the electronic patient dossiers of Shanghai Sixth People’s Hospital.

Tissue and pus samples were analyzed with the mNGS technology, completed by a third company (Genoxor Medical Technology, Shanghai, China). Among the 38 subjects, single samples of tissue or pus were collected in 20 patients, and double samples, including tissue and pus, were obtained in 18 patients (**Supplementary Table 1**). Therefore, 56 samples from the 38 patients were tested for pathogenic identification by mNGS. Meanwhile, 32 blood and 37 pus samples from 38 patients (**Supplementary Table 1**) were applied to the clinical microbial culture in our hospital.

### Sequencing and data analysis

In the mNGS analysis, DNA was extracted directly from tissue and (or) pus using the TIANamp Maxi DNA Kit (DP710, Tiangen Biotech, Beijing, China), following the manufacturer’s standard protocols. The extracted DNA was fragmented ultrasonically to yield 200-300bp fragments. DNA libraries were constructed through an end-repaired adapter and amplified through PCR. Agilent 2100 system (Agilent Technologies, Santa Clara, USA) was used for quality control of the DNA libraries, which were sequenced on the Illumina NextSeq platform (Genoxor Medical Technology, Shanghai, China). All sequencing data were deposited in the database under the Sequence Read Archive (SRA) accession number PRJNA827921.

High-quality sequencing data were generated after filtering out low-quality, low-complexity, and shorter reads (<35 bp) by bcl2fastq2. To eliminate the effect of the human sequences, the data mapped to the human reference genome were excluded using a powerful alignment tool called Bowtie2. The remaining data were aligned to the Microbial Genome Databases, including viruses, bacteria, fungi, and parasites. The databases were downloaded from NCBI (ftp://ftp.ncbi.nlm.nih.gov/genomes/). It contains 13434 whole-genome sequences of viral taxa, 7982 bacterial genomes or scaffolds, 917 fungi related to human infection, 4411 viruses related to human infection, and 124 parasites associated with human diseases.

### Interpretation of metagenomic data

The number of unique alignment reads was calculated and standardized to get the number of reads stringently mapped to pathogen species, standardized strictly mapped reads number (SDSMRN). In the report interpretation process, it needs to filter the blacklist and the negative control and analyze the specificity of the sequencing sequence. The blacklist is an in-house database. For different types of microbes, the thresholds were set as follows: Bacterial/Fungus: SDSMRN≥3, species was reported; Parasite: SDSMRN≥50; *Mycobacterium tuberculosis* complex (MTC)/Brucella/Nocardia: SDSMRNG≥1, species were reported.

### Statistical analyses

Histopathological results were accurate indications of infection or lack thereof and were regarded as the gold standard. Following the extracted data, 2×2 contingency tables were derived to determine sensitivity, specificity, positive predictive value (PPV), and negative predictive value (NPV). Sensitivity and specificity were calculated based on the formulas TP (true positive)/(TP+FN) (false negative) and TN (true negative)/(TN+FP) (false positive), respectively. PPV is expressed by the TP/(TP+FP) ratio, while NPV from the TN/(TN+FN). McNemar’s test was used to compare the sensitivity, specificity, PPV, and NPV. Comparison between two groups was analyzed using Fisher’s exact test, and a P<0.05 was defined as statistical significance. All statistical analyses were performed using SPSS 20.0 (IBM, Chicago, USA).

## Results

### Demographic characteristics and mNGS sequencing information

A total of 38 patients diagnosed with spinal infection were enrolled between January 1st, 2021 and December 31st, 2021, including 29 males and 9 females. The mean age was 57.4 ± 12.9 (ranges 23-71) years old. Patients with spinal infections commonly present with unspecific symptoms, such as back pain, fever, paresis, and contingent neurological deficits, which hinder early diagnosis. The laboratory test results are listed in **Table 1**. All the infectious patients underwent percutaneous transforaminal endoscopic debridement and drainage, and the histopathological analysis images were demonstrated in **Supplementary Figure 1**. The most common pathologic characteristics are bone necrosis, neutrophil infiltration, caseous necrosis, and tubercles. The culture result for mycobacteria was presented in **Supplementary Figure 2**. The information on the types of spinal infections was provided in **Table 1**, with one or more types in each patient. Vertebral osteomyelitis occurs in most cases, frequently combined with discitis, paravertebral infection, and epidural abscess. Statistically, Vertebral osteomyelitis was found in 35 (92.11%) cases, discitis in 20 (52.63%) cases, paravertebral infection in 11 (28.95%) cases, and epidural abscess in 2 (5.26%) cases. At the last follow-up (three months after surgery), all 38 patients recovered from the spinal infection.

**Table 1:** Demographic characteristic of the patients with spinal infection.

Characteristics		
Total number	Cases	38
Age,	Years, mean (range)	57.4 ± 12.9 (23-71)
Sex	Male (%)	29 (76.32)
	Female (%)	9 (23.68)
Laboratory tests	Hemoglobin (g/L)	121.6 ± 17.7
	WBC (×10^9^/L)	7.05 ± 4.32
	ESR (mm/h)	67.24 ± 34.34
	CRP (mg/L)	38.99 ± 62.16
Types of procedures	Percutaneous transforaminal endoscopic debridement and drainage (n, %)	38 (100%)
Types of spinal infection	Vertebral osteomyelitis (n, %)	35 (92.11%)
	Discitis (n, %)	20 (52.63%)
	Paravertebral infection (n, %) Epidural abscess (n, %)	11 (28.95%) 2 (5.26%)
Follow-up time	Months after treatment	3
Outcomes	Recovery (n, %)	38 (100%)

WBC, white blood cell; ESR, erythrocyte sedimentation; CRP, C-reactive protein.

### Pathogenic microorganisms identified by mNGS

**Figure 1:**
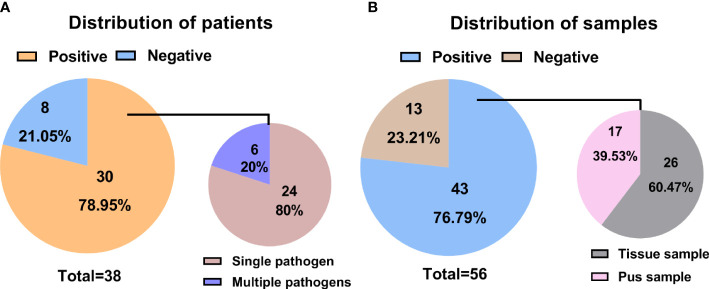
Statistical data on mNGS information in different patients and samples. **(A)** Distributions of positive and negative results in patients with spinal infection; **(B)** Distributions of positive and negative results in samples associated with a spinal infection.

**Figure 2:**
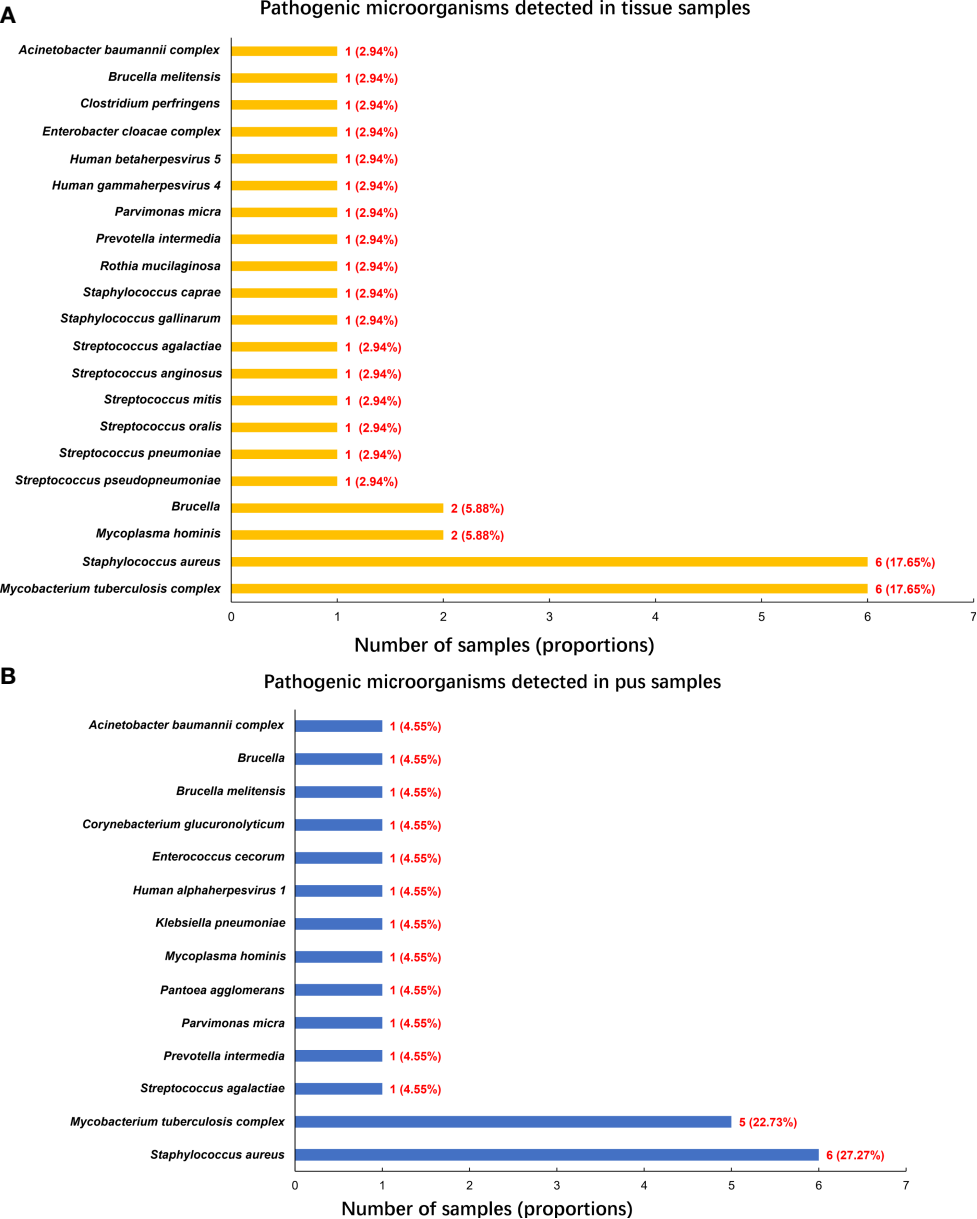
Pathogenic microorganisms detected by mNGS. **(A)** Pathogenic microorganisms detected in tissue samples; **(B)** Pathogenic microorganisms detected in pus samples.

A single tissue sample was collected in 16 patients, and single pus was collected in 4 patients. 18 patients performed a double-sample examination, including tissue and pus samples. To summarize, 34 tissue and 22 pus samples, a total of 56 samples from the 38 participants, were analyzed by mNGS. Following the optimized procedures, we conducted the mNGS analysis on the 56 samples, and a total of 19,735,816 raw reads were generated from sequencing, ranging from 3,014,099 to 51,271,214 reads per specimen. Of the 38 patients, there were 30 patients (78.95%) with positive mNGS results (**Table 2**), a single microorganism was detected in 24 (80%) patients, and multiple pathogens were found in 6 (20%) patients (**Figure 1A**). In the 56 samples, 43 (76.79%) were proven to be mNGS positive, including 26 tissue (60.47%) and 17 pus (39.53%) samples (**Figure 1B**).Pathogenic microorganism analyses revealed that 26 kinds of pathogenic microorganisms were identified by mNGS, among which 25 were classified into species. Still, *Brucella* in three samples was identified just at the genus level. 21 bacterial pathogens, one mycoplasma, and three viruses were recognized at the species level. *Human beta-herpesvirus 5*, *human herpesvirus 1*, and *human gammaherpesvirus 4* were separately identified in three samples. Statistically, 14 gram-positive and 7 gram-negative bacterial species were detected, adding *Brucella* as gram-negative. In the 34 tissue samples, the most frequent species detected through mNGS were *M. tuberculosis complex* (17.65%) and *S. aureus* (17.65%), followed by the *M. hominis* (5.88%) and *Brucella* (5.88%) (**Figure 2A**). Similarly, *S. aureus* (27.27%) was the top microorganism found in the 22 pus samples, and the second top was the *M. tuberculosis complex* (22.73%) (**Figure 2B**).

**Table 2:** Comparison of the positive rate of mNGS (with tissue and pus samples) and microbial culture (with blood and pus samples).

Methods	Cases	Positive cases	Negative cases	Positive rate	P value
mNGS	38	30	8	78.95%	
Microbial culture	38	17	21	44.74%	0.0042

### Comparison of mNGS versus microbial culture in pathogenic detection

**Figure 3:**
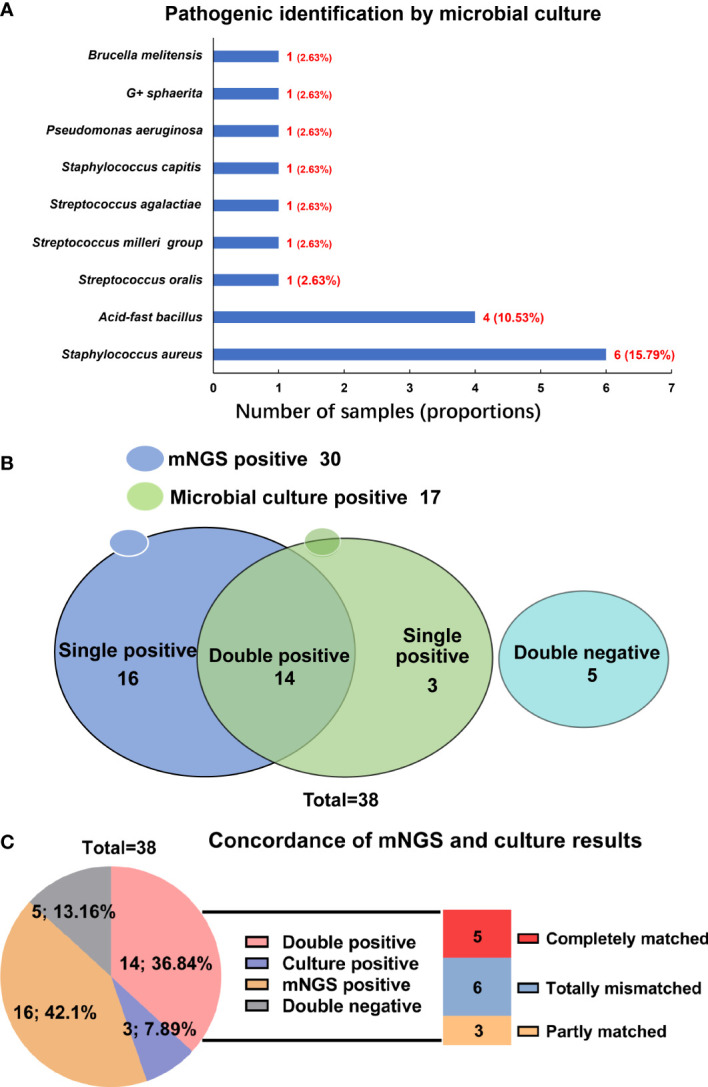
Comparison of mNGS and microbial culture in pathogenic identification in spinal infection. **(A)** Pathogenic microorganisms identified by microbial culture; **(B)** The detecting results of mNGS and microbial culture in different cases; **(C)** The concordance of mNGS and microbial culture in detecting pathogenic microorganisms.

In the present work, the microbial culture was the most used method for pathogenic identification in infectious diseases. Therefore, to evaluate the diagnostic performance of mNGS in spinal infection, the results of mNGS were compared to those of microbial culture. Among 38 patients, 32 blood and 37 pus samples were sent for microbial culture, once a positive result was yielded in either type of the sample, the patient would be judged as a positive result by microbial culture. However, culture-positive results were only found in 17 cases, with a positive rate of 44.74%. The most common microorganism identified by microbial culture was also the *S. aureus* (15.79%), followed by the *acid-fast bacillus* (10.53%) (**Figure 3A**).

When comparing results of mNGS with microbial culture, we compared the tissue and pus samples (for mNGS) with blood and pus samples (for microbial culture), and a significant difference was found between the positive rate by mNGS (78.95%) and by microbial culture (44.74%) (P=0.0042) (**Table 2**). In the 21 patients with pus samples for both mNGS and microbial culture, we evaluated the diagnostic performance of these two methods. As shown in **Table 3**, the sensitivity and specificity of mNGS were 84.2% and 100.0%, respectively, and these values of culture were 42.1% and 100.0%, separately.

**Table 3:** Comparison of diagnosis performance of mNGS and microbial culture in patients who had pus samples for both methods.

Methods	Cases	Sensitivity	Specificity	PPV	NPV
mNGS	21	84.2%	100.0%	100.0%	40%
Microbial culture	21	42.1%	100.0%	100.0%	15.38%

PPV, Positive predictive value; NPV, Negative predictive value.

**Figure 3B** indicated that the results of mNGS and microbial culture were both positive in 14 of 38 (36.84%) patients and both negative in 5 (13.16%) patients. 16 (42.11%) patients were determined to be single-positive by mNGS, and 3 (7.89%) were single-positive by microbial culture (**Figure 3B**). The information of the 14 double-positive cases was analyzed and presented in **Figure 3C**. **Table 4** showed the detailed pathogenic microorganisms detected by the two methods and their causative agents. In detail, 5 cases were completely matched (patients 1-5), while 6 cases were totally mismatched (patients 6-11). The remaining three patients (patients 12-14) were found to be “partly matched” defined as at least one overlap of pathogens when polymicrobial results were observed (**Figure 3C**).

**Table 4:** Matching of pathogenic microorganisms detected by mNGS and microbial culture.

Patients	mNGS	Microbial culture	Causative agent
Patient 1	*S. aureus*	*S. aureus*	*S. aureus*
Patient 2	*S. aureus*	*S. aureus*	*S. aureus*
Patient 3	*B. melitensis*	*B. melitensis*	*B. melitensis*
Patient 4	*S. aureus*	*S. aureus*	*S. aureus*
Patient 5	*S. agalactiae*	*S. agalactiae*	*S. agalactiae*
Patient 6	*M. tuberculosis complex*, *R. mucilaginosa*	*Acid-fast bacillus*	*M. tuberculosis*
Patient 7	*M. tuberculosis complex*	*Acid-fast bacillus*	*M. tuberculosis*
Patient 8	*S. caprae*	S. capitis	*S. caprae*, S. capitis
Patient 9	*M. tuberculosis complex*	*Acid-fast bacillus*	*M. tuberculosis*
Patient 10	*M. tuberculosis complex*	*Acid-fast bacillus*	*M. tuberculosis*
Patient 11	*S. mitis*, *S. oralis*, *S. pneumoniae*, *S. pseudopneumoniae*	*S. milleri group*	*Streptococcus*
Patient 12	*S. aureus*, *S. anginosus*	*S. aureus*	*S. aureus*
Patient 13	*S. aureus*, *H. betaherpesvirus 5*	*S. aureus*	*S. aureus*
Patient 14	*S. aureus*, *K. pneumoniae*, *E. cecorum*, *P. agglomerans*, *H. alphaherpesvirus 1*	*S. aureus*	*S. aureus*

### mNGS-guided antimicrobial therapy and clinical outcomes

According to the results of pathogenic identification, 33 patients were classified as infected, and 5 patients were considered aseptic. The pathogen identification results were applied to medication guidance. The target pathogens were confirmed accordingly for patients with double-positive results by mNGS and culture (14 patients), and the corresponding antibiotics were administrated. Otherwise, in patients with single positive results by mNGS (16 patients) or microbial culture (3 patients), a suspected pathogenic microorganism was proposed according to the comprehensive consideration based on clinical symptoms, and empiric antibiotic therapy was applied. If the outcome were desirable after drug use, the suspected pathogen would be confirmed to be the causative microorganisms. Three months later, all 38 patients were followed-up, and they experienced favorable outcomes, and no adverse effect was noted, proving the success of the mNGS-guided antimicrobial therapy.

### Evaluation of the optimal sample types for mNGS pathogenic detection

To find out the optimal sample types for mNGS pathogenic detection, we compared the diagnosis efficiency of tissue and pus samples. **Table 5** showcased that 26 were found to be positive in 34 tissues, and the positive rate was 76.47%. In the 26 positive samples, the causative agent pathogen was identified in 25 tissues; hence the accordance rate was 96.15% (25/26). Further, the positive rate in the 22 pus samples was 77.27%, and the accordance rate was 100%. There was no significance in the positive rate and accordance rate between the two types of samples (P>0.05). Overall, no significant superiority was noted between tissue and pus as the pathogenic detection sample by mNGS.

**Table 5:** Comparison of the diagnosis efficiency of tissue and pus samples by mNGS.

Sample types	Total number	Positive rate	Accordance rate
Tissue	34	76.47% (26, 8)	96.15% (25, 1)
Pus	22	77.27% (17, 5)	100% (17, 0)
P value		>0.05	>0.05

## Discussion

At present, mNGS provides the potential for fast pathogen identification without a prior target hypothesis. In the present study, we described the utility of mNGS in detecting the pathogenic organisms associated with spinal infection. A broad spectrum of pathogens in 25 species was seen, ranging from bacteria, viruses, to *M. hominis*. Meanwhile, the diagnostic performance of mNGS in spinal infection was compared with the traditional microbial culture pairwisely. It can be concluded that mNGS is superior to microbial culture, thereby emerging as a promising technique in the etiological examination of spinal infection.

Notably, *S. aureus* was our study’s most frequent bacterial pathogen identified by mNGS in 6 tissues and 6 pus samples. As a common pathogenic bacterium for spinal infection, *S. aureus* can cause a range of diseases, which has been reported to reduce antibiotic and immune cell penetration, leading to persistent and refractory infection (Gurtman et al., 2019; Gordon et al., 2020). Additionally, *M. tuberculosis* was our cohort’s second most frequent microorganism, which has been considered the world’s leading infectious killer (Chin, 2019). Spinal tuberculosis accounts for approximately 1% to 3% of all tuberculosis cases and 50% of musculoskeletal infections (Wang et al., 2021). It is worth noting that the opportunistic pathogen *M. hominis*, which causes the potential infection in the human genitourinary tract (Saadat et al., 2020), has not been reported in spinal infection. Fortunately, with the extensive use of mNGS technology, the rapid diagnosis and effective treatment of spinal infection caused by these microorganisms will be achieved soon.

By assessing the positive rate, species diversity, sensitivity and specificity, and its benefit to antimicrobial therapy, our data fully demonstrated that mNGS performed better than microbial culture in several aspects. Primarily, mNGS provided a higher pathogen detection ratio than standard culture-based testing, with a sensitivity of 84.2% in the pus samples. Another recent study has revealed 70.3% of sensitivity by mNGS in spinal infection tissue samples (Ma et al., 2022), which has been reported to range from 36% to 100% in diverse samples or under special status (Miao et al., 2018). Furthermore, our study proved that mNGS allowed a broader range of pathogen detection, and the underestimated polymicrobial infection might significantly benefit from it. Except for *M. tuberculosis*, its superior feasibility in identifying *Brucella* spp. has been demonstrated in the present study. In previous research, mNGS has been confirmed with a favorable effect in detecting *Brucella* spp. (Mongkolrattanothai et al., 2017). Given the high pathogenicity of these microorganisms, mNGS may contribute a lot in providing reference to clinical diagnosis. Nevertheless, the relatively high cost of mNGS is the main shortcoming that limits its sample size and hinders its wide use in clinical, a challenge that urgently needs to be addressed in the future.

Many may recognize the advantages of mNGS of shortened turnaround time (Gu et al., 2021). In addition, diverse clinical sample types allowed by mNGS are also essential for effective treatment. The analyses of the samples associated with spinal infection are more complicated than those from common lesion sites because of the thick pyogenic fluid specimens and fat-rich tissues (Di Martino et al., 2019). After analyses of the pathogens detected in tissue and pus samples by mNGS, no significant superiority was noted between them. The relatively small sample sizes could explain it, or the tissue and pus samples are all suitable candidates for mNGS testing in pathogen identification in spinal infection.

In summary, mNGS is certified to identify a broad spectrum of pathogenic microorganisms in patients with spinal infection. It performs better than the conventional microbial culture method in detection ratio, allowing for the identification of rare and vital pathogens and diverse samples. These findings highlight the critical role of mNGS in guiding drug administration and improving clinical outcomes for patients with a spinal infection. Moreover, the wide use of mNGS in pathogen detection can be expected soon.

## Data availability statement

The datasets presented in this study can be found in online repositories. The names of the repository/repositories and accession number(s) can be found in the article/**Supplementary Material**.

## Ethics statement

The studies involving human participants were reviewed and approved by the Ethics Committee of the Shanghai Jiao Tong University Affiliated Sixth People’s Hospital (No. 2017-127). The patients/participants provided their written informed consent to participate in this study.

## Author contributions

XL and XhC conceived of and designed the study, and provided data acquisition. YZ and JC collected the clinical data, analyzed and interpreted the data, and drafted the manuscript. XY contributed to the data analysis and interpretation, and the manuscript writing. ZC performed the study design, data collection, and manuscript drafting. TY and ZT contributed to the development of methodology and manuscript review and revision. GZ is a major contributor to data analyses and manuscript revision. XjC provided mNGS data acquisition and manuscript writing. All authors read and approved the final manuscript.

## Funding

This work was supported by grants from the National Natural Science Foundation of China (No. 82070615).

## Conflict of interest

XY and XjC are employed by the Genoxor Medical Science and Technology Inc.

The remaining authors declare that the research was conducted in the absence of any commercial or financial relationships that could be construed as a potential conflict of interest.

## Publisher’s note

All claims expressed in this article are solely those of the authors and do not necessarily represent those of their affiliated organizations, or those of the publisher, the editors and the reviewers. Any product that may be evaluated in this article, or claim that may be made by its manufacturer, is not guaranteed or endorsed by the publisher.

## Supplementary material

Supplementary Figure 1 Images show the histopathological examination of spinal infection. **(A)** The blue circle indicates the tubercles, and the yellow arrows indicate the Langhans multinucleated giant cells; **(B)** The red circles indicate caseous necrosis; **(C)** The blue circles indicate the granulomatous tubercles; **(D)** The red circle indicates caseous necrosis, and the yellow arrows indicate the Langhans multinucleated giant cells.

Supplementary Figure 2 Image indicates the culture results for mycobacteria.
