# Supplementary figures and images for: Evaluation of the metagenomic next-generation sequencing performance in pathogenic detection in patients with spinal infection
Source: Front Cell Infect Microbiol. 2022 Oct 27;12:967584. doi: 10.3389/fcimb.2022.967584 (PMC9646980; doi:10.3389/fcimb.2022.967584)

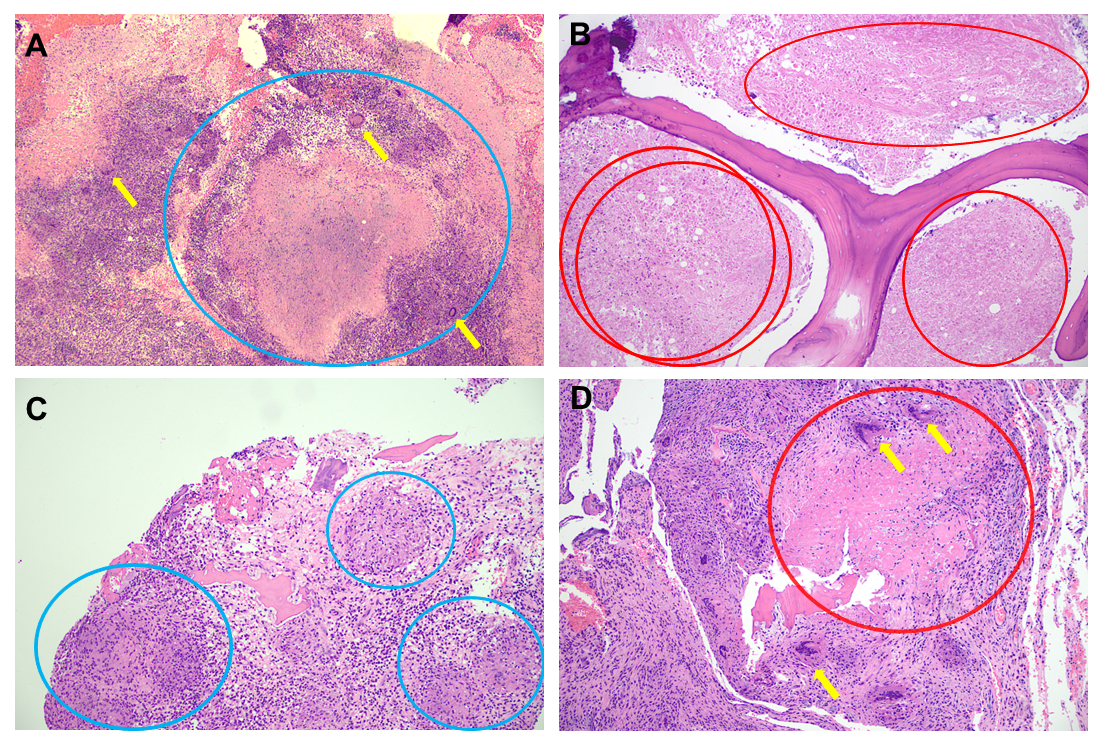

Supplement: Supplementary Figure 1 — Images show the histopathological examination of spinal infection. (A) The blue circle indicates the tubercles, and the yellow arrows indicate the Langhans multinucleated giant cells; (B) The red circles indicate caseous necrosis; (C) The blue circles indicate the granulomatous tubercles; (D) The red circle indicates caseous necrosis, and the yellow arrows indicate the Langhans multinucleated giant cells. [file Image_1.tif]

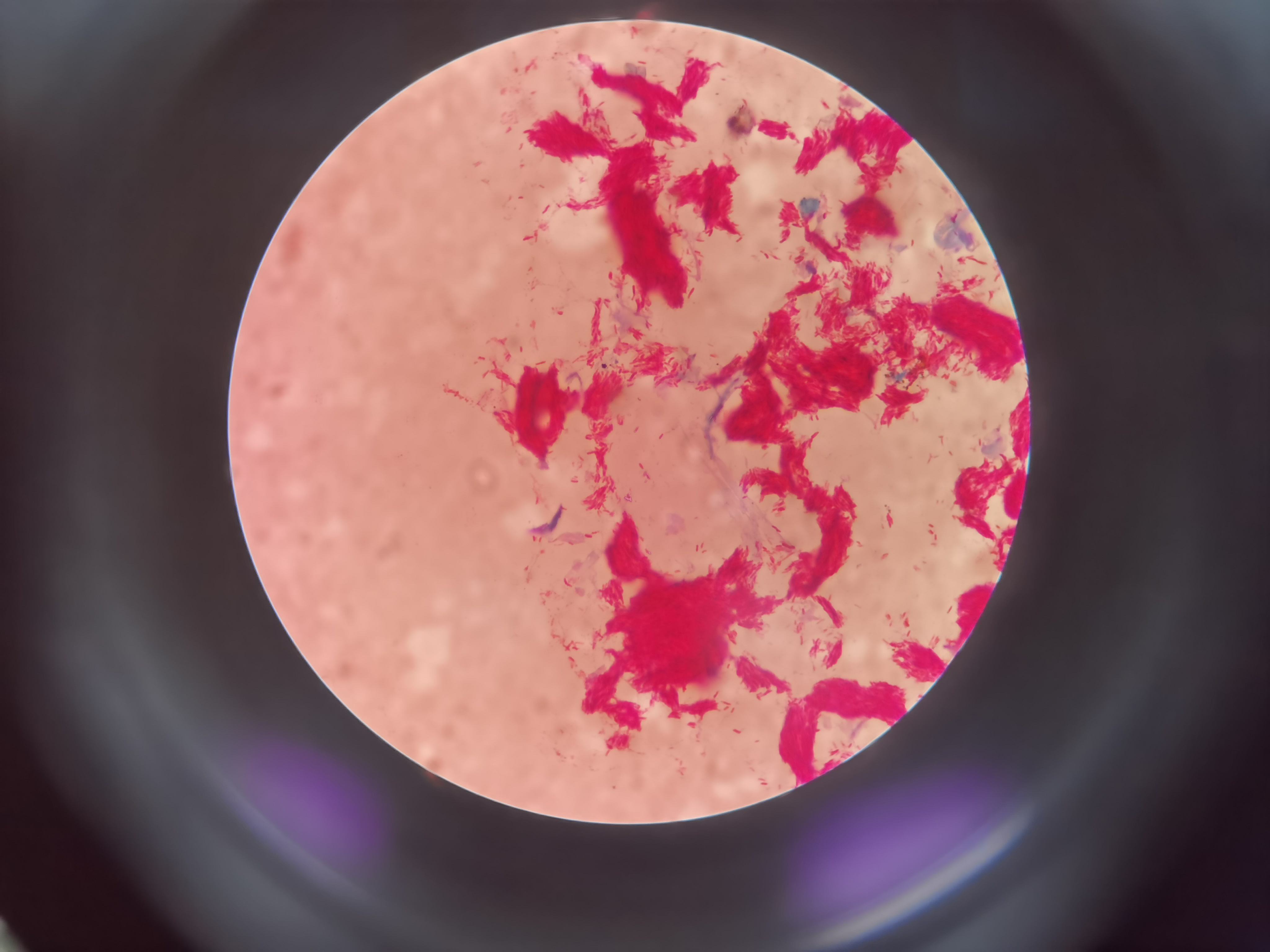

Supplement: Supplementary Figure 2 — Image indicates the culture results for mycobacteria. [file Image_2.tif]
