# Supplementary material for: Evaluation of the metagenomic next-generation sequencing performance in pathogenic detection in patients with spinal infection
Source: Front Cell Infect Microbiol. 2022 Oct 27;12:967584. doi: 10.3389/fcimb.2022.967584 (PMC9646980; doi:10.3389/fcimb.2022.967584)
Supplement: Supplementary file 3 [file Table_1.docx]

Supplementary Table 1 Detailed information of samples performed by mNGS and microbial culture

| Patients | mNGS | | Microbial culture | | Causative agent |
| --- | --- | --- | --- | --- | --- |
|  | Sample types | Results | Sample types | Results |  |
| Patient 1 | Pus | *S. aureus* | Pus  Blood | *S. aureus*  *S. aureus* | *S. aureus* |
| Patient 2 | Pus | *S. aureus* | Pus  Blood | *S. aureus*  *S. aureus* | *S. aureus* |
| Patient 3 | Pus  Tissue | *B. melitensis*  *B. melitensis* | Pus  Blood | *B. melitensis*  *B. melitensis* | *B. melitensis* |
| Patient 4 | Pus  Tissue | *S. aureus*  *S. aureus* | Blood | *S. aureus* | *S. aureus* |
| Patient 5 | Pus  Tissue | *S. agalactiae*  *S. agalactiae* | Pus  Blood | *S. agalactiae*  *S. agalactiae* | *S. agalactiae* |
| Patient 6 | Pus  Tissue | *M. tuberculosis complex*  *M. tuberculosis complex*, *R. mucilaginosa* | Pus | *Acid-fast bacillus* | *M. tuberculosis* |
| Patient 7 | Pus  Tissue | *M. tuberculosis complex*  *M. tuberculosis complex* | Pus | *Acid-fast bacillus* | *M. tuberculosis* |
| Patient 8 | Tissue | *S. caprae* | Pus  Blood | Negative  *S. capitis* | *S. caprae*, S. capitis |
| Patient 9 | Pus | *M. tuberculosis complex* | Pus  Blood | *Acid-fast bacillus*  Negative | *M. tuberculosis* |
| Patient 10 | Tissue | *M. tuberculosis complex* | Pus | *Acid-fast bacillus* | *M. tuberculosis* |
| Patient 11 | Tissue | *S. mitis*, *S. oralis*, *S. pneumoniae*,  *S. pseudopneumoniae* | Pus  Blood | Negative  *S. milleri group* | *Streptococcus* |
| Patient 12 | Tissue | *S. aureus*, *S. anginosus* | Pus  Blood | *S. aureus*  *S. aureus* | *S. aureus* |
| Patient 13 | Tissue | *S. aureus*, *H. betaherpesvirus 5* | Pus  Blood | Negative  *S. aureus* | *S. aureus* |
| Patient 14 | Pus  Tissue | *S. aureus*, *K. pneumoniae*, *E. cecorum*, *P. agglomerans*, *H. alphaherpesvirus 1*  *S. aureus* | Pus  Blood | *S. aureus*  Negative | *S. aureus* |
| Patient 15 | Tissue | *Brucella* | Pus  Blood | Negative  Negative | *Brucella* |
| Patient 16 | Tissue | Negative | Pus  Blood | Negative  *S. oralis* | *Streptococcus oralis* |
| Patient 17 | Tissue | *M. hominis* | Pus | Negative | *M. hominis* |
| Patient 18 | Tissue | *M. tuberculosis complex* | Pus  Blood | Negative  Negative | *M. tuberculosis complex* |
| Patient 19 | Pus | *S. aureus* | Pus  Blood | Negative  Negative | *S. aureus* |
| Patient 20 | Tissue | Negative | Pus  Blood | Negative  Negative | Negative |
| Patient 21 | Tissue | Negative | Pus  Blood | Negative  Negative | Negative |
| Patient 22 | Tissue | *S. aureus* | Pus | Negative | *S. aureus* |
| Patient 23 | Tissue | *C. perfringens* | Pus  Blood | Negative  Negative | *C. perfringens* |
| Patient 24 | Tissue | Negative | Pus | Negative | *M. tuberculosis complex* |
| Patient 25 | Tissue | *M. tuberculosis complex* | Pus  Blood | Negative  Negative | *M. tuberculosis complex* |
| Patient 26 | Tissue | Negative | Pus  Blood | Negative  *G+ coccus* | Negative |
| Patient 27 | Pus  Tissue | Negative  Negative | Pus  Blood | Negative  Negative | *M. tuberculosis complex* |
| Patient 28 | Pus  Tissue | *M. tuberculosis complex*  *M. tuberculosis complex* | Pus  Blood | Negative  Negative | *M. tuberculosis complex* |
| Patient 29 | Pus  Tissue | *S. aureus*  *S. aureus* | Pus  Blood | Negative  Negative | *S. aureus* |
| Patient 30 | Pus  Tissue | *M. hominis*  *M. hominis* | Pus  Blood | Negative  Negative | *M. hominis* |
| Patient 31 | Pus  Tissue | *A. baumannii complex*, *M. tuberculosis complex*  *A. baumannii complex* | Pus  Blood | Negative  Negative | *A. baumannii complex*, *M. tuberculosis complex* |
| Patient 32 | Pus  Tissue | *P. intermedia, C. glucuronolyticum*  *P. intermedia, S. gallinarum* | Pus  Blood | Negative  Negative | *P. intermedia* |
| Patient 33 | Pus  Tissue | Negative  Negative | Pus  Blood | Negative  *P. aeruginosa* | *P. aeruginosa* |
| Patient 34 | Pus  Tissue | Negative  *E. cloacae complex* | Pus  Blood | Negative  Negative | *E. cloacae complex* |
| Patient 35 | Pus  Tissue | *Brucella*  *Brucella* | Pus  Blood | Negative  Negative | *Brucella* |
| Patient 36 | Pus  Tissue | Negative  *H. gammaherpesvirus 4* | Pus  Blood | Negative  Negative | Negative |
| Patient 37 | Pus  Tissue | *P. micra*  *P. micra* | Pus  Blood | Negative  Negative | *P. micra* |
| Patient 38 | Pus  Tissue | Negative  Negative | Pus  Blood | Negative  Negative | Negative |
